# Application of ultrasound and microencapsulation on *Limosilactobacillus reuteri* DSM 17938 as a metabolic attenuation strategy for tomato juice probiotication

**DOI:** 10.1016/j.heliyon.2022.e10969

**Published:** 2022-10-05

**Authors:** Irene Giordano, Jumana Abuqwider, Mohammad Altamimi, Rossella Di Monaco, Sharon Puleo, Gianluigi Mauriello

**Affiliations:** a Department of Agricultural Science, University of Naples Federico II, 80049 Naples, Italy; b Department of Nutrition and Food Technology, Faculty of Agriculture and Veterinary Medicine, An-Najah National University, Nablus, Palestine

**Keywords:** Probiotic, Functional food, Tomato juice, Ultrasound, Microencapsulation

## Abstract

Counteracting probiotic-induced physicochemical and sensory changes is a challenge in the development of probiotic beverages. The aim of the study is to apply ultrasound and microencapsulation for the attenuation of *Limosilactobacillus reuteri* DSM 17938 to avoid change in a probiotic tomato juice. Preliminarily, six ultrasound treatments were applied. Probiotic survival in acid environment (pH 2.5) and bile salts (1.5 g/l) after ultrasound treatment was also studied. The probiotic was inoculated in tomato juice in four forms: free cells (PRO-TJ), sonicated-free cells (US-TJ), untreated-microencapsulated (PRO-MC-TJ) and sonicated-microencapsulated cells (US-MC-TJ). Probiotic viability and pH were monitored during 28 days of storage at 4 and 20 °C. Sensory analysis was performed for PRO-TJ and US-MC-TJ sample (4 °C). Ultrasound (57 W for 6 min) did not affect cell survival and transitorily modulated probiotic acidifying capacity; it reduced probiotic survival in acidic environment but increased probiotic survival in bile salts solution. Ultrasound was effective in maintain pH value of tomato juice but only at 4 °C. Instead, microencapsulation with sodium-alginate leads to a more stable probiotic juice, particularly at 20 °C. Finally, probiotication slightly modified some sensory attributes of the juice. This study shows the potential of ultrasound and microencapsulation as attenuation strategies and highlights the need for process optimization to increase ultrasound efficacy.

## 1 Introduction

In the recent years, there has been a change in diet worldwide. This trend is the result of the awareness acquired by the components of the agri-food system, industry and consumer, about the impact of food on human health. Conscious of the efficacy of a balanced diet in maintaining health and reducing the risk of certain diseases, the search for new foods and new food components was launched (Pimentel et al., 2021). Functional foods designate, as established by the European Commission, products that, beyond their nutritional properties, manifest beneficial effects on one or more body functions. This expression appears for the first time in Japan in 1980 as part of the "Systemic Analysis and Development of Food Functions" project. It is a broad category that includes products that naturally possess functional components and others that can be functionalized through technological interventions. Within this macro-category, probiotic foods have a significant position, in accordance with the knowledge acquired about the correlation between the composition of the gastrointestinal microbiota and human health (Olvera-Rosales et al., 2021).

The most recent definition of probiotic microorganisms was proposed by the International Scientific Association for Probiotics and Prebiotics (Hill et al., 2014). Probiotics are live microorganisms which, if taken in adequate amount, provide beneficial effects to the host. Both as a supplement and in food matrices, the most widely used probiotics belong to the group of Lactic Acid Bacteria (LAB) and to the genus *Bifidobacterium*. Recently, the taxonomy of *Lactobacillus*, based on phenotypic, genotypic and ecological factors, has been revised. Four new genera have been identified from the genus *Lactobacillus*: *Lacticaseibacillus*, *Lactiplantibacillus*, *Ligilactobacillus* and *Limosilactobacillus* (Zheng et al., 2020).

Traditionally, probiotic foods belong to the dairy food category. However, more and more subjects guided by ethical or endogenous factors, such as intolerances, allergies, predisposition to cardiovascular or cancer diseases, start vegetarian or vegan diets. Plant beverages, such as fruit and vegetable juices and aqueous extracts of cereals, legumes, nuts, seeds and pseudo-cereals have been extensively evaluated as potential carriers of probiotics (Pereira and Rodrigues, 2018; Valero-Cases et al., 2020). The probiotication of these beverages offers, on the one hand, the opportunity for food companies to become competitive in one of the most innovative research areas in the food sector; on the other hand, the possibility to meet the demands and the needs of different consumer groups.

Fruit and vegetable juices are excellent vehicles of probiotics as they are characterized by regular consumption and are appreciated by a heterogeneous group of consumers. Together with the naturally present bioactive components, such as minerals, vitamins, fibers and polyphenols, it is possible to add value through functionalization by probiotication. The main challenges in the production of probiotic juices are: to guarantee the survival and functionality of probiotic; to control the impact of the probiotic culture on the physico-chemical properties, sensory profile and general acceptance by the consumer (Pimentel et al., 2019; Rodríguez et al., 2021).

Through several approaches, properly called attenuation strategies, it is possible to control microbial cells performances (Bevilacqua et al., 2017). Commonly, attenuation has been applied to starter cultures used in cheese production, with the main aim of accelerating the ripening phase (Klein and Lortal, 1999; Madkor et al., 2000; El Soda et al., 2000; Di Cagno et al., 2012; Calasso et al., 2020). However, in more recent research attenuation has been proposed as a tool to avoid or slow down the fermentation by microorganisms and the consequent acidification (Bevilacqua et al., 2016; Racioppo et al., 2017; Campaniello et al., 2020). In the era of non-thermal technologies, sonication represents a means to counteract this phenomenon. Although microencapsulation is extensively used to improve cell survival during incorporation of probiotics into food and after ingestion, it can also be considered as an attenuation strategy. In fact, depending on the polymer used, the microencapsulation technique and the matrix-polymer interactions, the microcapsule acts as a physical barrier and as a selective permeable membrane thus controlling the interactions between the probiotic and the external environment. To incorporate microcapsules in foods is necessary to satisfy some safety criteria. Typically, biopolymers, such as polysaccharides and proteins, are widely used as encapsulation materials, alone or in combination with each other. Among these, alginate is the most commonly used for probiotic encapsulation. Biocompatibility, bioavailability, edibility, cheapness, GRAS (Generally Recognized As Safe) status make it suitable for the development of an oral transport system (Yao et al., 2020).

The aim of this work was to evaluate the potential of sonication to control *Limosilactobacillus reuteri* DSM 17938 metabolism and the effect of the treatment on its functionality. Furthermore, combined microencapsulation and sonication were investigated as an attenuation strategy for the formulation of a probiotic tomato juice. Along a 28 days storage at 4 and 20 °C, the viability, pH and the probiotic influence on the sensory properties were evaluated.

## 2 Materials and methods

### 2.1 Microorganism and culture conditions

In this study *Li*. *reuteri* DSM 17938, kindly provided by BioGaia (BioGaia AB, Stockholm, Sweden), was used as a probiotic. The culture was stored at −18 °C in MRS broth (OXOID Ltd., Basingstoke, Hampshire, England) with the addition of glycerol (SIGMA, Milan, Italy). Before each test, the strain was cultured in MRS broth at 37 °C for 24 h.

### 2.2 Culture sonication

The ultrasound treatment was applied through the LABSONIC U (B. Braun) equipment. The sonicator is equipped with different probes and in this experiment the 50 ml cup probe was used. Before and after sonication, the probe was cleaned with 70% ethanol.

The protocol was developed based on a previous study (Racioppo et al., 2017) with some modifications. Cells from a fresh culture of *Li*. *reuteri* DSM 17938 (30 ml) were harvested by centrifugation (ALC® Centrifuge PK130, Thermo Fisher Scientific) at 6000 g for 10 min and subsequently resuspended in an equal volume of sterile deionized water. The main parameters of sonication step were power level, duration time and duty cycle (the percentage of time during which the ultrasound signal is “on”). Preliminarily, a total of six treatments were set up: 57 W-4 min, 57 W-6 min, 64 W-4 min, 64 W-6 min, 74 W-4 min, 74 W-6 min. The duty cycle was set at 50% and remained unchanged for all applications. Each treatment was evaluated based on the attenuation of the fermentative metabolism. Briefly, the sonicated cell suspension was inoculated in MRS broth (1% inoculum) and incubated at 37 °C. The pH was monitored using a digital pH-meter (BENCH METER-pH 80) after 6 and 24 h of incubation. The untreated microorganism was used as a control and data collected reported as pH decrease (ΔpH). Furthermore, viable count was assessed, before and after ultrasound treatment, on MRS Agar. The plates were incubated at 37 °C for 48 h and results expressed as Log CFU/ml. Data collected from the viable count and attenuation were used to select the treatment to be further investigated.

### 2.3 Effect of ultrasound on functional properties

The effect of ultrasound on *Li*. *reuteri* DSM 17938 functional properties was tested. In this experiment, the ability of the probiotic to survive at low pH and in the presence of bile salts was evaluated. The pellet of the sonicated cell suspension at 57 W-6 min and of the untreated microorganism was resuspended in: i) acidified sterile deionized water (pH 2.5); ii) a solution of bile salts (1.5 g/l) (Oxoid). Following an incubation period of 3 h at 37 °C, viable count was performed as described above.

### 2.4 Microencapsulation

Untreated and sonicated (57 W for 6 min) probiotic cells were microencapsulated. Microencapsulation was carried out through the Encapsulator B-395 Pro (BÜCHI Labortechnik, Flawil, Switzerland). The vibration technique of encapsulation was derived from De Prisco et al. (2015). Sodium alginate with medium viscosity (Sigma, product n. A2033) solution (12 g/l) was prepared in deionized water. In detail, a total volume of 50 ml of the cell suspension was harvested by centrifugation at 6000 g for 15 min, washed twice with Quarter Strength Ringer solution and then suspended in equal volume of the sodium alginate solution. The alginate-cell suspension was load into a sterile syringe. The microencapsulator was fed, through the syringe, with a flow rate of 3.20 ml/min. The vibration frequency and the electrode voltage were respectively set at 1300 Hz and 1800 mV. Through a 120 μm nozzle, the droplets of alginate-cell suspension were collected in a gelling bath of a 0.5 mol/l CaCl_2_ solution under constant stirring. The sedimented microcapsules were recovered and the supernatant was removed.

### 2.5 Probiotic tomato juice formulation

A pasteurized tomato juice was purchased from a local supermarket. Four probiotic formulations were prepared: PRO-TJ, tomato juice added of untreated microorganism in free form; US-TJ, tomato juice added of sonicated cells in free form; PRO-MC-TJ, tomato juice added of microcapsules of untreated cells; US-MC-TJ, tomato juice added of microcapsules of sonicated cells. The cell load of 10^7^ CFU/ml of *Li. reuteri* DSM 17938 was reached in all formulations.

### 2.6 Storage conditions, viability ad pH value

Tomato juice formulations were distributed in 40 ml aliquots, stored at 4 and 20 °C for 28 days and monitored for probiotic viability and pH. Sampling times were 0, 14 and 28 days for the refrigerated juice and 0, 5, 10, 15, 20 and 28 days for the juice stored at 20 °C. To count the encapsulated bacteria, first decimal dilution was carried out in 9 ml of 0.2 M sterile sodium citrate solution to promote disgregation of microcapsules and cell leakage.

### 2.7 Sensory evaluation

PRO-TJ and US-MC-TJ juices stored at 4 °C were chosen for the sensory evaluation. Sensory evaluation was carried out at the end of storage time by using the “Difference-from-Reference” method. Therefore, one sample was designated the “Reference” and all the other samples were compared with the Reference in terms of degree of difference. Among the samples, a blind reference was used to measure the reliability of the panel.

Sensory panel was composed of 30 semi-trained assessors (20 women and 10 men) ranging in age from 20 to 58 (average 31 ± 7.8 y.o.).

In detail, during the test, 4 samples (15 ml per sample) were presented to each assessor at refrigeration temperature (±4 °C): the reference, (commercial tomato juice), a sample of PRO-TJ, a sample of MC-TJ and a blind reference. Each sample was marked with a random three-digit numeric code, except for the reference marked with "R". The assessors were asked to indicate whether there were differences between the samples and the reference through 10 cm linear scale, ranging from 0 (equal to the reference) to 10 (completely different from the reference). The assessors were also informed that some of the test samples may be the same as the reference sample. Finally, they were asked to list the attributes that discriminate the samples. Data collection was performed using “Fizz Acquisition” software (Biosystémes, Counternon, France).

The scores achieved by all the samples during the difference from control test were submitted to one sample t-test (P ≤ 0.05) to evaluate whether they were significantly different from the reference.

The study was conducted in agreement with the guidelines of the Declaration of Helsinki and the Italian ethical requirements on research activities and personal data protection (D.L. 30.6.03 n. 196). Signed informed consent was obtained from all subjects involved in the study in double copy. One copy was returned to the subject, while the other one was kept by the researcher in charge. The written informed consent included the aim of the study, the risks resulting from the participation in the study (the subjects were informed that the sample preparation would have been conducted according to the “good clinical practice”, ART. 6:2, D.Lg. 24.06.2003 n°211, Dir. 2001/20/CE), the possibility to withdraw the consent without any justification and the list of ingredients and allergens.

### 2.8 Statistical analysis

All the analyses were repeated three times (n = 3) and results were reported as the mean of the experiments performed with the standard deviation. Data were analysed through two-way ANOVA and Bonferroni test (SPSS-Software) to ascertain significant differences between the means. Significance was declared at P < 0.05.

## 3 Results and discussion

### 3.1 Viability and acidifying capacity of attenuated strain

The effectiveness of ultrasound was assessed exposing the cells to six power-time combinations. Tables 1 and 2 reported the results of preliminary sonication experiments. After 6 h of incubation a reduction in acidification occurred. By increasing the power and/or the duration of the treatment, the attenuation effect was higher. However, after 24 h no significant differences (P > 0.05) between the tested combinations and the control were observed. Furthermore, the treatments at 64 and 78 W for 6 min were lethal for the probiotic. The population load reduced by 1.61 and 2.94 Log CFU/ml, respectively. Instead, at 57 W for 4 and 6 min, 64 W and 78 W for 4 min the decrease in viability was less than 1 Log CFU/ml. Among these, the combination 57 W-6 min determines the lowest decrease in pH (0.08, t_6_). Therefore, this combination was chosen and used in the further evaluations.

**Table 1:** Decrease (mean value ±standard deviation, n = 3) of *Li. reuteri* DSM 17938 population (N_0_ = 9.26 ± 0.20 Log CFU/ml) after treatment at different ultrasound combinations.

Ultrasound treatments	ΔLog CFU/ml
57 W for 4 min	0.27 ± 0.05^a^
64 W for 4 min	0.52 ± 0.08^a^
78 W for 4 min	0.59 ± 0.16^a^
57 W for 6 min	0.36 ± 0.04^a^
64 W for 6 min	1.61 ± 0.22^b^
78 W for 6 min	2.94 ± 0.10^c^

Different letters within the same time of treatment indicate a significant difference between mean values as determined by a T-test (P < 0.05).

**Table 2:** **-** Decrease (mean value ±standard deviation, n = 3) of pH of MRS broth inoculated at 1% with a culture of *Li. reuteri* DSM 17938 previously treated by ultrasound at different conditions.

Ultrasound treatments	Time of incubation at 37 °C	
	6 h	24 h
57 W for 4 min	0.63 ± 0.18^a^	2.04 ± 0.04^a^
64 W for 4 min	0.73 ± 0.08^a^	2.01 ± 0.08^a^
78 W for 4 min	0.25 ± 0.06^b^	2.10 ± 0.16^a^
57 W for 6 min	0.08 ± 0.03^a^	2.04 ± 0.03^a^
64 W for 6 min	0.03 ± 0.01^a^	2.03 ± 0.08^a^
78 W for 6 min	0.00 ± 0.01^a^	2.06 ± 0.13^a^

Different letters, within the same time of incubation and treatment, indicate a significant difference between mean values as determined by T-test (P < 0.05).

The effects induced by ultrasound on microbial cells are associated with physical and chemical factors related to the phenomena of cavitation and acoustic flow. The data collected showed clearly a transient effect of ultrasound treatment for all tested combinations. This means that cell can resuscitate, that is a damage the cell can successfully repair. Based on this, to understand the mechanism of action of ultrasound it may be necessary to investigate the functionality of the sugar transport systems, as well as of enzymes and proteins involved in the basic functions for growth. The results obtained confirmed those of the study by Racioppo et al. (2017). Similarly, testing different combinations of sonication power/duration on different strains, the attenuation was temporary. As suggested in the aforementioned study, multiple interventions could guarantee a prolongation of attenuation. Briefly, they stored the untreated and the sonicated cells at 4 and 15 °C to achieve a longer lasting effect. Although at refrigeration temperatures there were no significant differences (P > 0.05) in the acidification of the culture media, at 15 °C the behaviour of the sonicated strains was different from the control, showing a significant (P < 0.05) lower acidification.

### 3.2 Effect of ultrasound on functional properties

The assessment of *Li. reuteri* DSM 17938 functionality, before and after sonication, was evaluated performing survival tests (Table 3). After 3 h of incubation at 37 °C in acidic environment (pH 2.5) the treated cells were more sensitive (P < 0.05) than the untreated ones with a reduction of 2.30 Log CFU/ml and 0.91 Log CFU/ml, respectively. Otherwise, sonication appears to improve *Li. reuteri* DSM 17938 resistance in the 1.5 g/l bile salts solution. The treated cells showed a remarkable survival (P < 0.05) with a reduction of 3.29 Log CFU/ml compared to that of the untreated cells of 6.12 Log CFU/ml.

**Table 3:** Decrease (mean value ±standard deviation, n = 3) of *Li. reuteri* DSM 17938 population (ΔLog CFU/ml) after 3 h of incubation at 37 °C under simulated gastrointestinal (GI) condition. US-TR cells were pre-treated at 57 W for 6 min.

Sample	GI condition	
	pH 2.5	0.15% bile salts
Control	0.91 ± 0.03^a^	6.12 ± 0.15^a^
US-TR	2.30 ± 0.12^b^	3.29 ± 0.04^b^

Different letters within the same condition indicate a significant difference between mean values as determined by T-test (P < 0.05).

The cell membrane is the first site where the effects of ultrasound can be observed. The mechanical stress induced by the collapse of the cavitation bubbles and by the high shear forces alters the structure of the membrane causing the destruction of the phospholipid bilayer. In addition, the localized increase in temperature and pressure lead to the formation of free radicals that react with polyunsaturated fatty acids, triggering peroxidation reactions (Yeo and Liong 2011). Overall, the physical destruction and alteration of the lipid composition modify the permeability of the membrane making the cell more sensitive to H^+^ ions. In several studies, the loss of membrane integrity in sonicated cells has been attested. Pore formation was observed by Yang et al. (2010) who showed images obtained by transmission electron microscope where the treated cells presented small holes. On the other hand, the structure was intact in fresh cells. Interesting, bigger holes occurred with a prolongated exposure, causing the leak of cytoplasm. In other studies, the increased permeability was indirectly measured with assays for the release of intracellular components. In the work of Bevilacqua et al. (2019), sonication leads to an increased absorption at 260 nm (nucleic acids) and at 280 nm (proteins) for *Propionibacterium freudenreichii* subsp. *freudenreichii* DSM 20271 and *Acidipropionibacterium jensenii* DSM 20535.

The resistance of some *Lactobacillus*, including different strains of *Li. reuteri* (Meleh et al., 2020; Puttarat et al., 2021; Martoni et al., 2008), towards bile salts is well documented. From the results obtained (Table 3) the *Li. reuteri* DSM 17938 increases resistance in presence of bile salts (0.15%) upon the treatment. However, the results suggest that ultrasound treatment has contrasting effects on its functional properties. Probably, the changes induced by sonication is strain depend. In the work of Racioppo et al. (2017) ultrasound did not modify *Li. reuteri* DSM 20016 survival in acidic environment (pH 2.0 and 2.5) and in presence of bile salts (3 g/l). Instead, the physical treatment affected *Lactiplantibacillus plantarum* L12 and *Bifidobacterium infantis* Bb02 in both solutions but the treated *Bifidobacterium longum* Bb46 resulted in a lower survival only in the bile salts solution.

### 3.3 Viability of probiotic during storage

**Figure 1:**
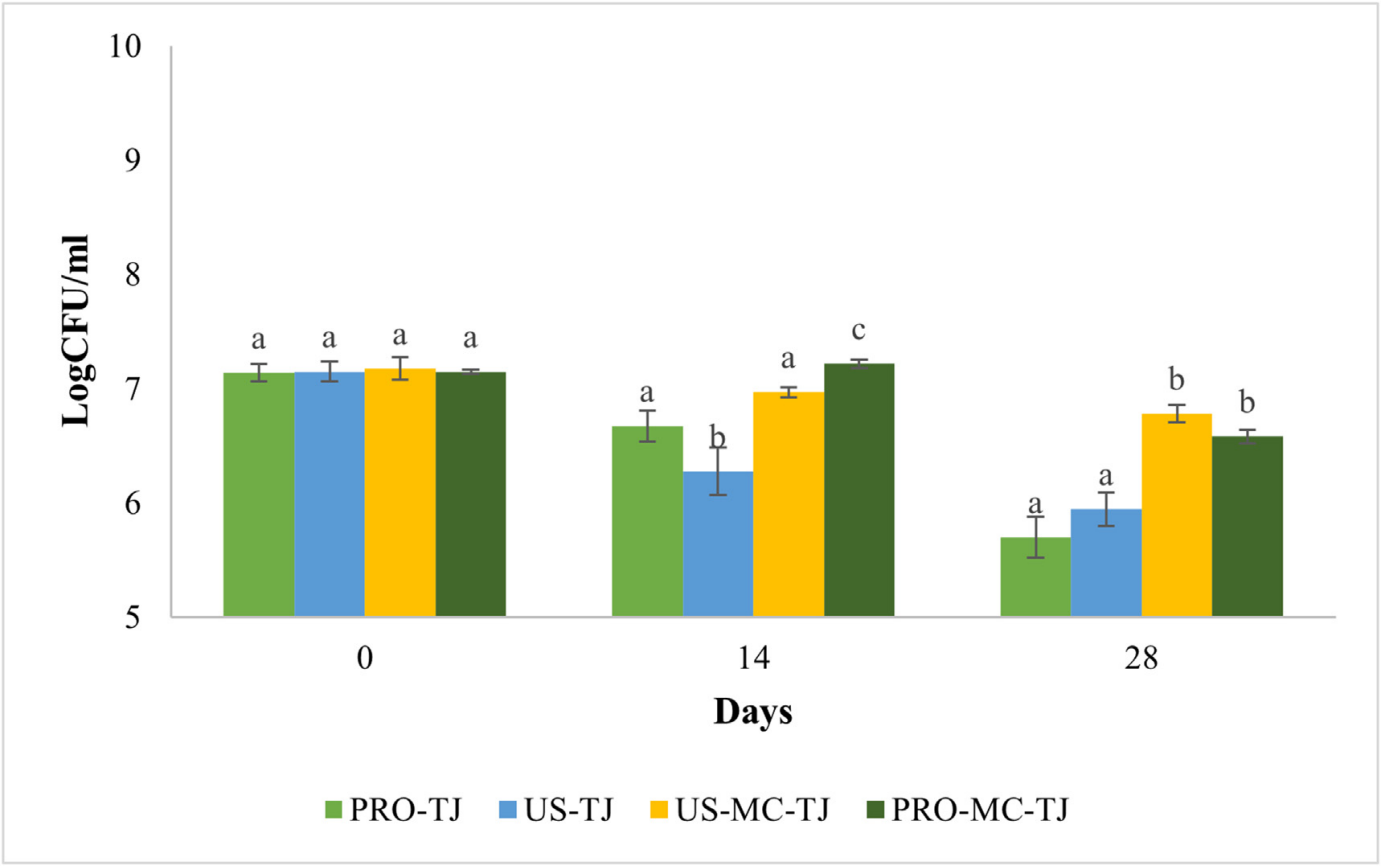
Viability of free-untreated (PRO-TJ), free-sonicated (US-TJ), sonicated-microencapsulated (US-MC-TJ) and untreated-microencapsulated (PRO-MC-TJ) Li. reuteri DSM 17938 in tomato juice during 28 days of storage at 4 °C. Values are reported as the means of three experiments ±standard deviation. For each storage time different letter indicates that the differences between the samples are significant (P < 0.05).

Free cells of *Li. reuteri* DSM 17938 sonicated and un-sonicated showed a similar trend in viability when added to tomato juice, at both 4 and 20 °C. At 4 °C during the first 14 days of storage, the microbial load in PRO-TJ sample was more stable and the reduction rate was faster over the remaining time. In US-TJ sample the opposite phenomenon occurred (Figure 1). Nonetheless, minimal differences in viability were observed. In fact, the viable numbers of probiotic were, at the end of the 4 weeks of storage, 5.70 for PRO-TJ and 5.94 Log CFU/ml for US-TJ. Even if the decrease from t_0_ is limited (1.44 and 1.21 Log CFU/ml for PRO-TJ and US-TJ, respectively), the values achieved are below the limits usually proposed for probiotic foods (10^6^–10^7^ CFU/g). Similar findings were reported in a study by Zhu et al. (2020) where three types of juice, apple, orange and tomato, were chosen for enrichment with *Lactobacillus sanfranciscensis*. A decline in probiotic viability in all three juices was observed. Despite the reduction, the viable count at the end of storage was in accordance with the recommended level for probiotic foods. Differently from our study the initial load was higher (8 Log CFU/ml). Probably, the behaviour of the probiotic is related to the low pH of the medium. Although LAB are acidophilic microorganisms, a prolonged exposure to acidic environment (pH ≤ 4.5) forces the cell to an energy surplus for maintenance of the physiological intracellular pH. Consequently, the removal of ATP for vital functions caused cell death. Generally, another factor that contributes to cell damage is exposure to O_2_. Pimentel et al. (2015a) investigated the influence of the type of packaging on *Lacticaseibacillus paracasei* ssp. *paracasei* viability in apple juice. They found that glass packages were more effective in maintain probiotic viability than the plastic ones. The key factor is the extremely low permeability of glass to the O_2_ compared to the polyethylene. Lycopene is an oxygen scavenger, so we can hypothesize that it could have a protective effect on probiotics during storage, thus promoting a more favourable anaerobic environment.

**Figure 2:**
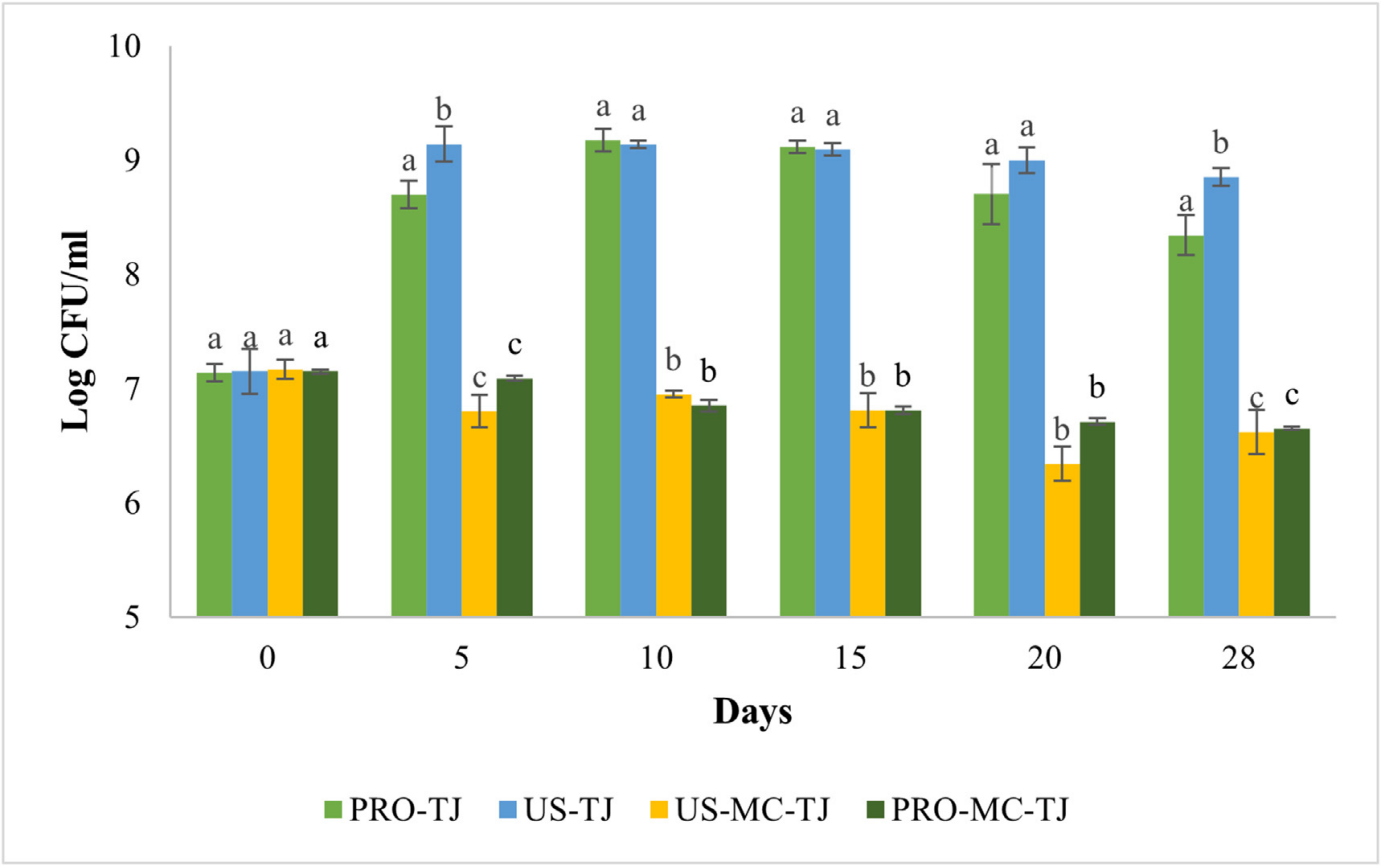
Viability of free-untreated (PRO-TJ), free-sonicated (US-TJ), sonicated-microencapsulated (US-MC-TJ) and untreated-microencapsulated (PRO-MC-TJ) Li. reuteri DSM 17938 in tomato juice during 28 days of storage at 20 °C. For each storage time different letter indicates that the differences between the samples are significant (P < 0.05).

In PRO-TJ and US-TJ samples in the first 5 days of storage at 20 °C there was a rapid increase in population load, then the viable numbers remain almost constant (Figure 2). Sonication did not lead to significant differences between the juice enriched with the probiotic in free form (P > 0.05), regardless of the storage temperature. Probably, probiotics are able to repair membrane injuries and restore their metabolism at higher temperatures (Campaniello et al., 2020). Moreover, LAB are spoilage microorganisms for tomato juice and so it is an ideal environment for their growth.

Microencapsulated samples were significant different in viability (P < 0.05) from the PRO- and US-TJ samples. Moreover, the most stable viability was found in both US-MC-TJ and PRO-MC-TJ samples, at both 4 and 20 °C (Figure 1 e 2). However, no significant differences (P > 0.05) were found between the two microencapsulated samples. This result confirms that sonication does not affect probiotic viability when added to tomato juice. It is well-known that microencapsulation keeps the cells alive and at the same time prevent them from growing. In the work of Gandomi et al. (2016) chitosan-alginate microencapsulation of *Lactobacillus rhamnosus* GG leads to 4.5 times greater survival rate during 90 days of storage in apple juice than the probiotic in free form. It is well established that microencapsulation is a very useful approach to guarantee probiotic survival in many food matrices (Frakolaki et al., 2021; Iqbal et al., 2021). The influence of microencapsulation on bacteria viability depends on polymer physical-chemical properties and the interaction with the food matrix. In the work of Olivares at al. (2919) *Lacticaseibacillus casei* DSM 20011 was added in pineapple, orange and raspberry juice. For each juice two probiotic formulations were tested: i) free form; ii) encapsulated form using sodium alginate. After 28 days of storage at 4 °C, the microcapsules recovered had 100% and 91% viability for pineapple and orange juice, respectively. Instead, in raspberry juice the viability disappeared. Probably, the phenomenon was caused by the high absorption of anthocyanin inside the microcapsules.

Therefore, the collected data and the reported studies show that probiotic viability is very sensitive and depends on the strain, the addition form, the type of juice and its compositions.

### 3.4 pH values during storage

**Figure 3:**
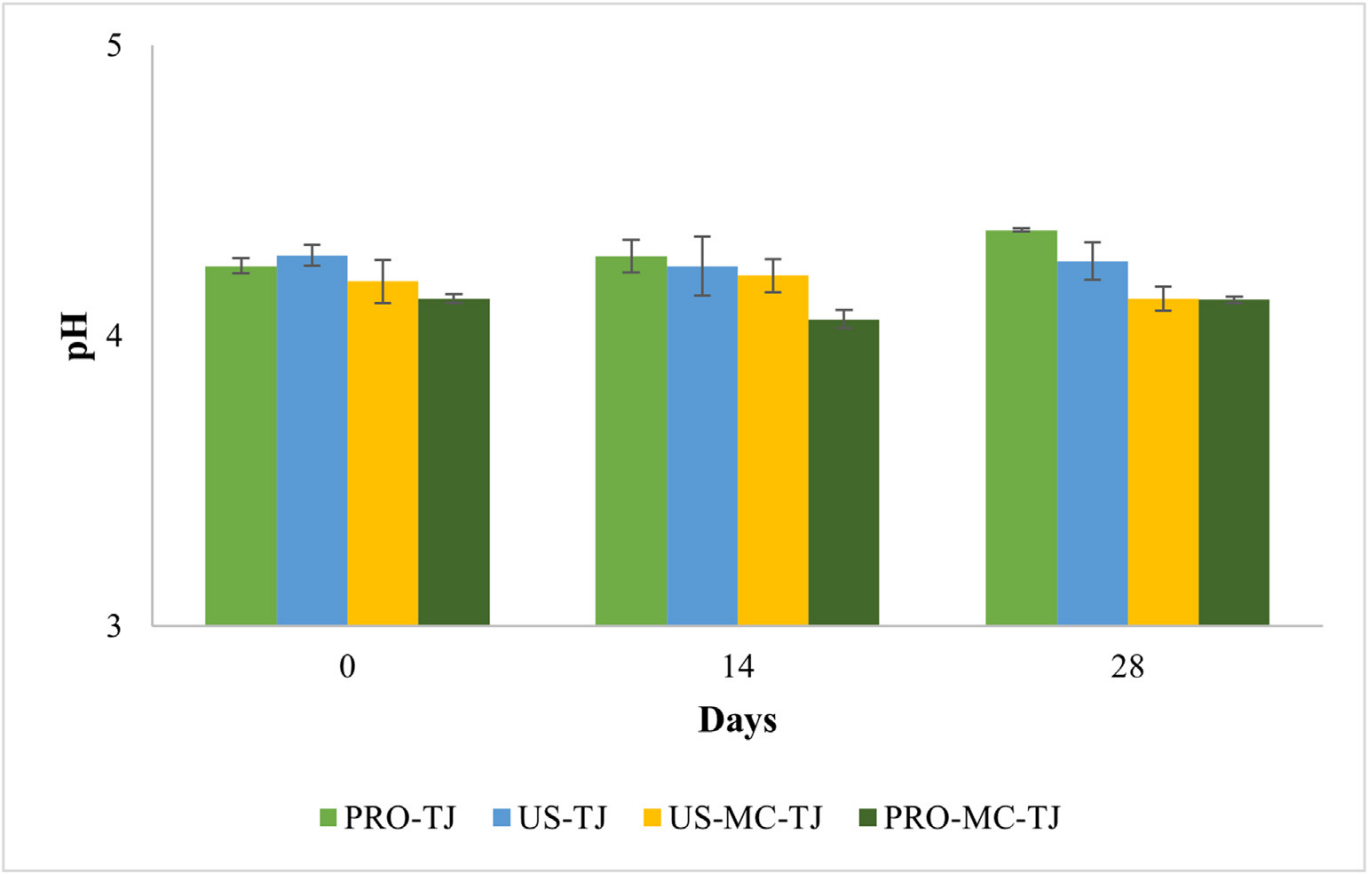
pH of tomato juice containing free-untreated (PRO-TJ), free-sonicated (US-TJ), sonicated-microencapsulated (MC-TJ) and untreated-microencapsulated (PRO-MC-TJ) Li. reuteri DSM 17938 during 28 days of storage at 4 °C. The differences were always not significant between the samples for each storage time.

**Figure 4:**
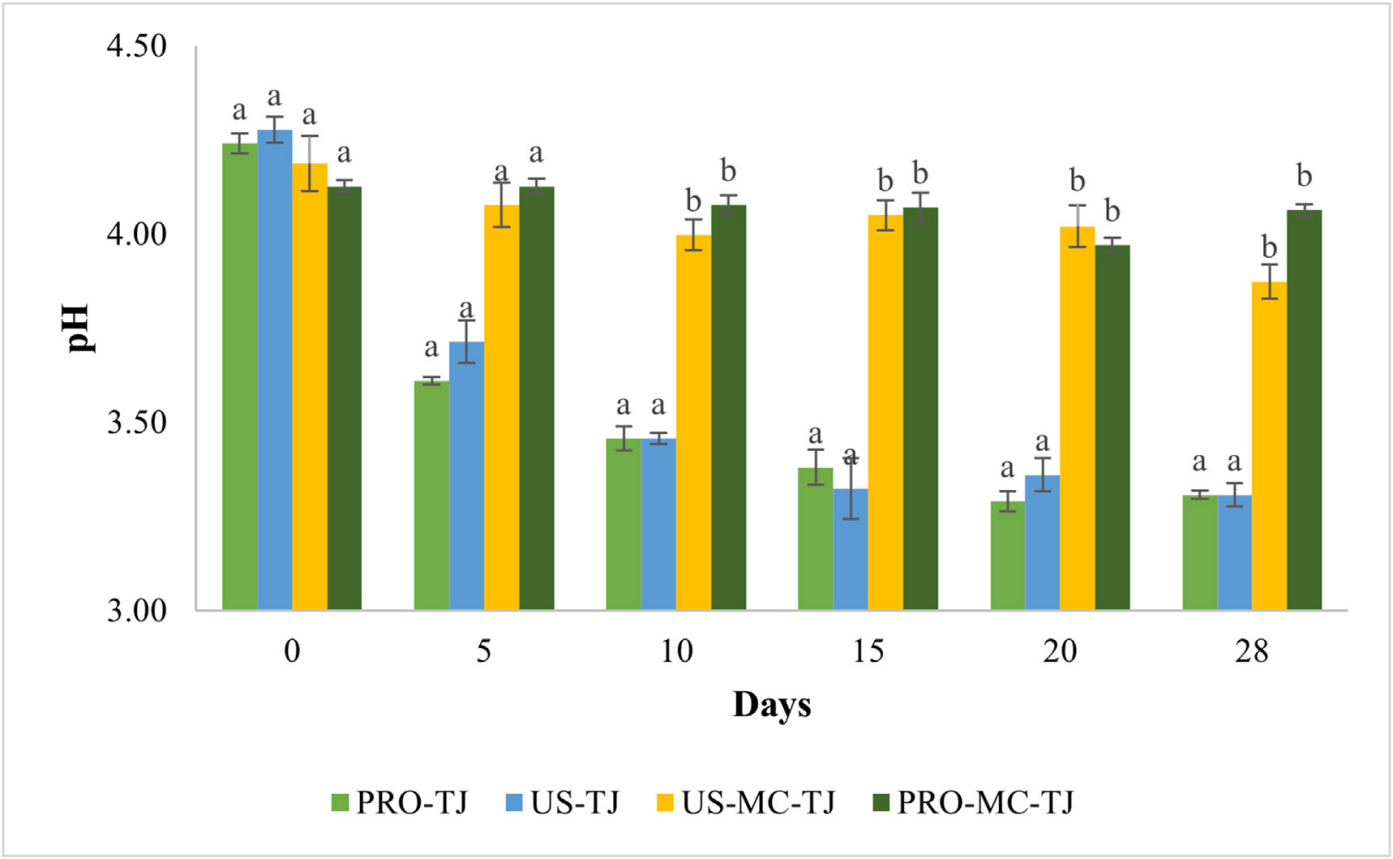
pH of tomato juice containing free-untreated (PRO-TJ), free-sonicated (US-TJ), sonicated-microencapsulated (MC-TJ) and untreated-microencapsulated (PRO-MC-TJ) Li. reuteri DSM 17938 during 28 days of storage at 20 °C. For each storage time different letter indicates that the differences between the samples are significant (P < 0.05).

No significant differences (P > 0.05) occurred in pH values of the refrigerated juices. The addition of the *Li. reuteri* DSM 17938 in free form caused a slight increase in pH over the time during the refrigerated storage (Figure 3), with the highest pH of 4.36. Potentially, the bacterial deaminase may have catalysed the release of amino groups from the protein of the juice (8 g/l). So, the increased pH could be the consequence of the increase in ammonium ions concentration. The US-TJ sample showed no changes in pH, since the values remained constant until the end of storage (ΔpH 0.02). Results regarding the effect of ultrasound on acidification of a probiotic beverage are in agreement with the findings of Campaniello et al. (2020). Sonication was applied on *Lactiplantibacillus plantarum* c16 and c19 to counteract probiotic acidification when inoculated in a rice beverage. The probiotic beverage was stored under thermal abuse before and then at 4, 15 e 20 °C. They found that ultrasounds were efficient at delay acidification only when combined with refrigeration temperature. In fact, as reported above, ultrasound cause reversible effect on bacteria membrane and the ability of the microorganism to restore their homeostasis is, probably, faster at higher temperature. So, it is possible to hypothesize a greater effectiveness of ultrasound when associated with treatments at low temperature as a strategy to attenuate the fermentative metabolism of probiotics in functional beverages. Even at 20 °C the PRO and US-TJ samples exhibit a similar trend in pH (P > 0.05) (Figure 4). Undoubtedly, this phenomenon is due to the strong growth of the probiotic.

In US-MC-TJ and PRO-MC-TJ, at both temperatures, minimal acidification occurred. Comparing with the juices supplemented with probiotics in free form and stored at 4 °C, encapsulation of sonicated and untreated *Li. reuteri* DSM 17938 did not lead to significant differences (P > 0.05) in pH. Instead, it significantly (P < 0.05) improved pH stability at 20 °C. However, US-MC-TJ is not statistically different (P > 0.05) from PRO-MC-TJ. Therefore, sonication does not contribute to *Li. reuteri* DSM 17938 attenuation. These results suggest that microencapsulation alone is an effective attenuation strategy for *Li. reuteri* DSM 17938 when incorporated in tomato juice. In fact, as a physical barrier, the microcapsule controls the uptake of nutrients from the external environment and the release of metabolites minimizing physical-chemical changes.

### 3.5 Sensory analysis

The sensory assessment of tomato juice was conducted on day 28. Commercial juice (the Reference) and two probiotic formulations, PRO-TJ and US-MC-TJ (4 °C) were compared to evaluate organoleptic change during the storage period. Scores (mean ± s.d.) for the blind reference, PRO-TJ and US-MC-TJ sample are reported in Table 4. The majority of the assessors (83%) recognized the blind reference. The differences between the blind reference and the PRO-TJ sample and between the blind reference and the US-MC-TJ sample were then analysed by Dunnett's test. Regardless of the addition form, probiotic juices were perceived significantly different (P < 0.05) from the commercial one.

**Table 4:** Sensory scores of the perceived differences through a scale ranging from 0 (equal to the reference) to 10 (completely different from the reference) between the reference and the samples.

Tester	Samples		
	Hidden reference	PRO-TJ	PRO-MC-TJ
1	0.7	10	6.7
2	6.7	6.7	6.7
3	0.1	9.2	8.9
4	0.0	8.1	9.3
5	0.0	2.7	1.5
6	0.0	8.3	7.1
7	0.4	6.9	5.0
8	9.8	0.1	5.0
9	8.2	8.6	7.8
10	0.0	5.4	4.4
11	0.9	8.1	10
12	0.0	8.7	3.2
13	4.1	4.8	5.8
14	0.2	5.0	9.8
15	0.0	0.9	1.7
16	0.3	4.7	5.7
17	0.4	1.7	7.4
18	8.0	8.0	0.0
19	0.2	0.2	2.4
20	0.0	6.7	2.1
21	0.0	2.6	1.5
22	0.0	5.1	2.8
23	2.1	7.8	2.0
24	0.3	8.4	9.0
25	8.5	7.0	0.1
26	0.0	2.9	5.0
27	0.2	5.5	8.3
28	0.0	5.0	9.1
29	0.1	9.5	7.1
30	0.0	10	10

In probiotic beverages production, to meet consumer's needs, it is pivotal to consider the impact of probiotic addition on sensory attributes and to understand which ones are affected by probiotication. Panellists indicated acidity, sweetness, viscosity and texture as the attributes that mainly differentiate the commercial juice from probiotic ones. Consumers exert different behaviour concerned probiotic juices. In some works, perceptible sensory differences were described between conventional and functional juices. In particular, the last is perceived more acidic and with medicinal and dairy taste compared to the traditional juice (Luckow and Delahunty, 2004a & b). In others, no appreciable changes in aroma, taste or general acceptability were found after probiotic addition (Perricone et al., 2014; Pimentel et al., 2015b; da Costa et al., 2017).

## 4 Conclusion

This study proved that sonication alone produces a temporary attenuation of fermentative metabolism of *Li. reuteri* DSM 17938 without affecting its viability. More findings are required to better understand the mechanism by which the phenomenon occurs. Concerning the probiotics traits, controversial results were obtained. Ultrasound treatment seems to reduce probiotic survival in acid environment and enhanced its resistance to bile salts. In addition, the tomato juice probiotication showed that it is a suitable medium for probiotic incorporation. Interesting, sonicated cells did not change juice pH under refrigeration (4 °C) storage. Unfortunately, sonicated strain showed a decline in viability at 4 °C. Microencapsulation improved probiotic viability during the refrigerated storage and maintained viable numbers and pH values of probiotic tomato juice at 20 °C. However, combining ultrasound technology with microencapsulation does not increase or change the attenuation effect induced by microencapsulation itself. The data collected from the sensory analysis, showed that *Li. reuteri* DSM 17938, regardless of the form of addition, affects gustatory, olfactory and mechanical attributes of a commercial tomato juice.

## Declarations

### Author contribution statement

Irene Giordano: Performed the experiments; Analyzed and interpreted the data; Wrote the paper.

Jumana Abuqwider; Sharon Puleo: Performed the experiments.

Mohammad Altamimi: Analyzed and interpreted the data.

Rossella Di Monaco; Gianluigi Mauriello: Conceived and designed the experiments.

### Funding statement

This research did not receive any specific grant from funding agencies in the public, commercial, or not-for-profit sectors.

### Data availability statement

Data will be made available on request.

### Declaration of interest’s statement

The authors declare no conflict of interest.

### Additional information

No additional information is available for this paper.
